# Directly measured secondhand smoke exposure and COPD health outcomes

**DOI:** 10.1186/1471-2466-6-12

**Published:** 2006-06-06

**Authors:** Mark D Eisner, John Balmes, Edward H Yelin, Patricia P Katz, S Katherine Hammond, Neal Benowitz, Paul D Blanc

**Affiliations:** 1 Department of Medicine, University of California, San Francisco, UCSF Box 0924, San Francisco, CA, 94113-0924, USA; 2 Division of Occupational and Environmental Medicine, Department of Medicine, University of California, San Francisco, UCSF Box 0924, San Francisco, CA, 94113-0924, USA; 3 Division of Pulmonary and Critical Care Medicine, Department of Medicine, University of California, San Francisco, UCSF Box 0924, San Francisco, CA, 94113-0924, USA; 4 Institute for Health Policy Studies, University of California, San Francisco, UCSF Box 0920, San Francisco, CA, 94113-0920, USA; 5 School of Public Health, University of California, Berkeley, USA; 6 Division of Clinical Pharmacology and Experimental Therapeutics, Department of Medicine, University of California San Francisco, UCSF Box 1220, San Francisco, CA, 94143-1220, USA

## Abstract

**Background:**

Although personal cigarette smoking is the most important cause and modulator of chronic obstructive pulmonary disease (COPD), secondhand smoke (SHS) exposure could influence the course of the disease. Despite the importance of this question, the impact of SHS exposure on COPD health outcomes remains unknown.

**Methods:**

We used data from two waves of a population-based multiwave U.S. cohort study of adults with COPD. 77 non-smoking respondents with a diagnosis of COPD completed direct SHS monitoring based on urine cotinine and a personal badge that measures nicotine. We evaluated the longitudinal impact of SHS exposure on validated measures of COPD severity, physical health status, quality of life (QOL), and dyspnea measured at one year follow-up.

**Results:**

The highest level of SHS exposure, as measured by urine cotinine, was cross-sectionally associated with poorer COPD severity (mean score increment 4.7 pts; 95% CI 0.6 to 8.9) and dyspnea (1.0 pts; 95% CI 0.4 to 1.7) after controlling for covariates. In longitudinal analysis, the highest level of baseline cotinine was associated with worse COPD severity (4.7 points; 95% CI -0.1 to 9.4; p = 0.054), disease-specific QOL (2.9 pts; -0.16 to 5.9; p = 0.063), and dyspnea (0.9 pts; 95% CI 0.2 to 1.6 pts; p < 0.05), although the confidence intervals did not always exclude the no effect level.

**Conclusion:**

Directly measured SHS exposure appears to adversely influence health outcomes in COPD, independent of personal smoking. Because SHS is a modifiable risk factor, clinicians should assess SHS exposure in their patients and counsel its avoidance. In public health terms, the effects of SHS exposure on this vulnerable subpopulation provide a further rationale for laws prohibiting public smoking.

## Background

Personal direct cigarette smoking is the most important single causal factor for developing COPD. The view that cigarette smoking is the *sole *meaningful factor in the epidemiology and natural history of COPD, however, is a misconception. Although direct cigarette smoking is the major cause of COPD, up to two cases out of ten cannot be attributable solely to this risk factor.[1] Other exposures, particularly secondhand smoke (SHS) exposure and occupational exposures, may be important in the development of COPD.[2-4] In terms of disease progression, other initiators of COPD besides direct smoking may also influence the course of the disease and its eventual health outcomes. Although SHS exposure among adults with COPD appears to be common, the effects of SHS have not been systematically examined in persons with established COPD.[5,6]

Because SHS contains many potent respiratory irritants, it is biologically plausible that exposure would adversely affect the clinical course of established COPD. Supporting this hypothesized causal relationship, it is known that SHS exposure has negative effects on adults with established asthma, including greater respiratory symptom severity, increased asthma medication use, impaired health-related quality of life, and more frequent hospitalizations for asthma.[7-11] Nonetheless, the specific effects of SHS exposure on COPD have not been elucidated.[5,6] We used data from a population-based prospective cohort study to examine the impact of directly measured current SHS exposure on COPD-related health status.

## Methods

### Overview

We used data from a population-based prospective cohort study of adults with COPD. SHS exposure was assessed using both self-report from structured telephone interviews and direct exposure assessment (urine cotinine and personal nicotine badge monitors). Using these exposure and outcomes data, we examined the longitudinal association between SHS exposure and health status among persons with COPD.

### Definition of COPD

We used the standard epidemiologic approach to define COPD based on a self-reported physician diagnosis of chronic bronchitis, emphysema, or COPD.[12-14] During the telephone interview, subjects were asked whether they had ever received a physician's diagnosis of any of several chronic respiratory conditions. Those who reported physician diagnoses of chronic bronchitis or emphysema were considered to have COPD, along with those who specifically reported a physician diagnosis of COPD. We included respondents with COPD who had concomitant asthma because they clinically resemble persons with COPD alone.[15] As reported previously, we validated the case definition of COPD using spirometry in a subgroup of 47 participants with COPD whose physicians provided spirometry reports (of 386 subjects).[2]

### Study recruitment

The study was approved by the University of California, San Francisco Committee on Human Research. We used data from wave 3 and wave 4 of a population-based multi-wave longitudinal cohort study of U.S. adults to elucidate the impact of SHS exposure on COPD health outcomes. Direct measures of SHS exposure were obtained only in these waves. Initial recruitment was previously reported in detail.[2,4]. Briefly, 2,113 adults aged 55 to 75 years were initially recruited by random digit dialing among residents of the 48 contiguous U.S. states with random over-sampling of geographic areas that had the highest published COPD mortality rates.[16] The random "hot spot" sample was further enriched by randomly over-sampling subjects with COPD. The initial overall study participation rate was 53% among households with an eligible respondent present and there were 383 subjects with COPD.

**Figure 1:**
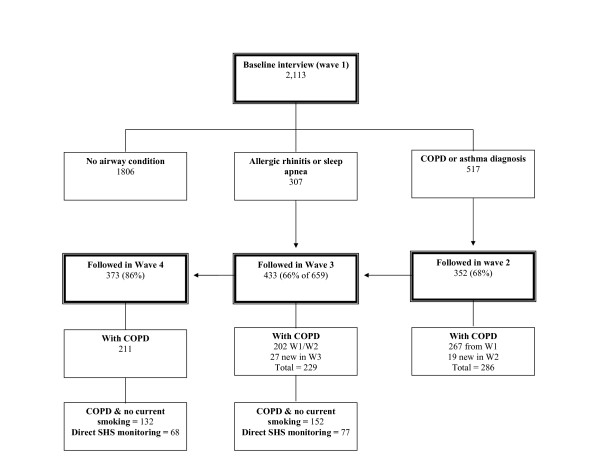
Details of sampling and recruitment for baseline through wave 4. Wave 2 attempted to recruit all subjects who indicated a diagnosis of asthma or COPD at baseline. Wave 3 attempted to recruit all subjects followed in wave 2 plus those who indicated a diagnosis of allergic rhinitis or obstructive sleep apnea at baseline. Wave 4 attempted to recruit subjects who completed wave 3 follow-up.

Details of the wave 2 follow-up interview have also been previously reported.[17] Approximately 12 months after the initial interview, we attempted to contact all 517 subjects who reported either COPD or asthma at baseline interview. Of these subjects, 352 (68%) completed the follow-up interview, of whom 267 reported COPD at baseline and an additional 19 indicated COPD at wave 2 (total 286) This follow-up and direct SHS monitoring participation (see below) is summarized in Figure 1.

At wave 3, which was approximately 12 months after wave 2, we attempted to contact all 352 subjects with asthma or COPD plus an additional 307 subjects who reported other airways diseases reported at baseline (allergic rhinitis and obstructive sleep apnea). Of the total 659 subjects, 433 completed the wave 3 interview (66%) and 229 subjects had COPD, which included 27 newly reported COPD cases at wave 3. Wave 4 was conducted about 12 months after wave 3, with completion of interviews in 373 of 433 subjects (86%), which included 211 respondents with COPD. There were no statistical differences in sociodemographcic characteristics (age, sex, race, educational attainment), health status (COPD severity score or SF-12 physical component summary score), or smoking status by follow-up status from wave 2 to wave 3 (p > 0.10 in all cases). Subjects who completed wave 4 were more likely to be white, non-hispanic (90% vs. 78%) and to have a college or graduate degree (30 vs. 12%) than were those who did not complete wave 4 follow-up (p < 0.05); there were no other statistical differences (p > 0.10 in all cases).

The current analysis was restricted to subjects who completed wave 3 and who reported no current smoking (either wave 3 or wave 4, if applicable) and had urine cotinine levels that were less than 100 ng/ml, a level which is consistent with direct personal smoking.[18-20] In sum, 152 current non-smoking subjects with COPD were eligible, of whom 77 subjects completed direct SHS monitoring with urine cotinine and personal badge nicotine measurements at baseline (wave 3), which corresponds to a 51% completion rate. Of this group, 68 subjects completed the direct SHS exposure follow-up approximately 1 year later (wave 4) (88% completion rate among direct SHS monitoring group). As shown in Table 1, there were no statistical differences between the 152 current non-smokers with COPD who did and did not complete baseline direct SHS measurement (p > 0.15, all cases). There were also no statistical differences between those who completed baseline direct SHS assessment who did and did not complete the 1 year follow-up assessment (p > 0.05, all cases).

### Structured telephone interviews

Participants completed structured telephone interviews that included health history, cigarette smoking, SHS exposure, sociodemographic characteristics, and health status. Direct personal cigarette smoking was evaluated using standard questions from the National Health Interview Survey. [21] Based on these responses, subjects were defined as current smokers, ex-smokers, and never smokers.

### Self-reported SHS exposure

We previously developed and validated a survey instrument that assesses recent ETS exposure.[8] The instrument, which was tailored for adults with asthma living in Northern California, assesses exposure during the past 7 days in 6 microenvironments: the respondent's home, another person's home, traveling in a car or another vehicle, workplace (including dedicated smoking areas), bars and nightclubs, and other locations. In each area, the instrument ascertains the total duration (in hours) of exposure during the past 7 days. Based on the distribution of responses, we defined three categories of exposure: no exposure, lower level exposure (1–3 hours/week), and higher level (≥4 hours/week) exposure.

### Direct SHS monitoring

We used a combined approach to conduct direct SHS exposure monitoring based on urine cotinine and personal nicotine badges. Cotinine, a metabolite of nicotine, is a widely used and specific biomarker of SHS exposure.[22] Cotinine has a short half-life of 20 hours and reflects shorter term SHS exposure than the personal nicotine badge that measures average 7-day exposures. Urine samples and completed badges were returned to the investigators by mail (completion rates are provided above).

Concentrations of cotinine and *trans*-3'-hydroxycotinine, which is the proximate metabolite of cotinine and the most abundant metabolite of nicotine present in urine, were determined using liquid chromatography-tandem mass spectrometry (LC-MS/MS).[23,24] The method is similar to a published method for determining cotinine concentrations in serum of non-smokers.[25] Deuterium-labeled cotinine (cotinine-d9) and deuterium-labeled *trans*-3'-hydroxycotinine (*trans*-3'-hydroxycotinine-d9) were used as internal standards. The mass spectrometer was operated in the positive ion mode using atmospheric pressure chemical ionization. Quantitation was achieved using selected reaction monitoring (SRM) of the transitions m/z 177 to m/z 80 for cotinine, m/z 193 to m/z 80 for *trans*-3'-hydroxycotinine and the transitions m/z 186 to m/z 84 and m/z 202 to m/z 84 for the respective internal standards. Limits of quantitation were 0.05 ng/mL for cotinine and 0.1 ng/mL for *trans*-3'-hydroxycotinine.

The extent of hepatic metabolism of nicotine to cotinine, which occurs primarily via cytochrome P450 2A6 (CYP2A6), and the rate of cotinine metabolism both determine the mathematical relationship between nicotine intake from SHS exposure and cotinine level.[23] There are substantial inter-individual differences in CYP2A6 activity, which could affect cotinine levels for a given nicotine dose. Because cotinine is also converted to *trans*-3'-hydroxycotinine via CYP2A6, the ratio of *trans*-3'-hydroxycotinine/cotinine is highly correlated with the clearance of nicotine.[23] Consequently, this marker can be used as a non-invasive marker of nicotine metabolism.

Each subject was instructed to wear the personal nicotine badge monitor during regular activities for 7 days. The passive monitor, which has been previously described, samples nicotine from ambient air.[26,27] A 4-cm-diameter polystyrene cassette holds a filter treated with sodium bisulfate and a membrane filter functions as a windscreen. Ambient nicotine diffuses to the treated filter, where it is trapped. The collected nicotine is analyzed by gas chromotography with nitrogen selective detection. Based on the amount of nicotine measured on the filter (ug), which represents the total amount of nicotine collected during the monitoring period, the nicotine concentrations were calculated by dividing the nicotine collected by the estimated volume of air sampled (monitoring duration multiplied by sampling rate of 24 ml/minute). The passive monitors have a limit of detection less than 0.01 ug per filter and a coefficient of variability of 0.11 for replicate analysis.[26] Based on the distribution of values, we divided urine cotinine and badge nicotine levels into tertiles for statistical analysis.

### Study outcome variables: COPD-related health status

We used a combined approach with disease-specific and generic health status measurements to assess COPD-related health status. To measure disease severity, we previously developed and validated a disease-specific COPD severity score for use in epidemiologic and outcomes research.[28] Based on survey responses, the COPD severity score is comprised of 5 overall aspects of COPD severity: respiratory symptoms, systemic corticosteroid use, other COPD medication use, previous hospitalization or intubation for respiratory disease, and home oxygen use. Each item was weighted based on clinical aspects of the disease and its expected contribution to overall COPD severity. Possible total scores range from 0 to 35, with higher scores reflecting more severe COPD.

Generic physical health status was measured with the SF-12 Physical Component Summary Score. The SF-12 is derived from the Medical Outcomes Study SF-36 instrument, which is the most widely used measure of generic health status. The SF-36 has been extensively validated in the general population[29] and among adults with COPD.[30] Defined from the eight SF-36 subscales by factor analysis, the physical component summary score reflects an underlying physical dimension of physical HRQL.[31] Higher scores reflect more favorable health states.

We used the Airways Questionnaire 20 (AQ-20) to measure disease specific quality of life.[32] This instrument is a short survey that was validated against the St. George's Respiratory Questionnaire, which is a 50 item instrument that has been used extensively in COPD to measure disease-specific QOL.[33,34] It has excellent psychometric properties for assessing QOL in COPD and asthma and higher scores correspond to poorer QOL.[32,35] Dyspnea was measured using three of five questions from the modified MRC dyspnea scale.[36] Higher scores indicate greater levels of dyspnea.

### Statistical analysis

Statistical analysis was conducted using SAS 9.1 (Cary, NC). Bivariate analysis was conducted using the unpaired t-test for continuous variables and likelihood ratio chi-square test for dichotomous variables. The analysis was conducted both for self-reported SHS exposure and directly measured SHS exposure (urine cotinine and personal badge nicotine). To ensure comparable results, the analysis was restricted to subjects who completed at least baseline direct SHS monitoring. We used linear regression analysis to examine the cross-sectional impact of SHS exposure and health-related COPD status (COPD severity, generic physical health status, disease-specific QOL, and dyspnea). We used multivariate linear regression analysis to control for variables that could confound the relationship between SHS exposure and health outcomes, including age, sex, race, educational attainment, and previous smoking history (all subjects were current non-smokers).[2,37] Furthermore, we used multivariate linear regression to elucidate the impact of baseline SHS exposure on COPD-related health status at 1 year follow-up. To take into account inter-individual differences in nicotine metabolism, we repeated the multivariate analysis after adding the ratio of *trans*-3'-hydroxycotinine/cotinine, a non-invasive measure of CYP2A6 activity, to the model.

To express the "clinical significance" of the impact of SHS on COPD-related health status, we expressed the change in each health status variable in terms of proportional change in standard deviation of the score. It has been shown that a one-half standard deviation in health status variables corresponds to the minimally important difference.[38] Consequently, we evaluated the impact of SHS exposure on each health status variable according to this criterion.

As above, participants in the direct SHS monitoring program were similar to non-participants, including socioeconomic status, COPD severity, and physical health status. To further take non-response into account, sampling weights were developed using all the personal characteristics in Table 1. The weighted analysis was not substantively different from the unweighted analysis, so we report the unweighted analysis only.

### Role of the funding source

The funding source was not involved in study design, data collection, statistical analysis, or manuscript preparation.

## Results

### Subject characteristics

The majority of subjects were white and female, with an average age of 64.4 years (Table 1). The cohort had a generally low educational attainment, with nearly half completing high school or less. Although all subjects were current non-smokers (see Methods), the majority indicated a previous history of smoking (72%). Baseline health status indicators are also provided in the Table 1.

### Self-reported SHS exposure and COPD-related health outcomes

Of the 77 non-smokers with COPD who completed direct SHS monitoring, 26% (95% CI 17 to 37%) indicated 1 or more hours of SHS exposure during the past 7 days. Table 2 shows the distribution of exposure for self-reported and directly measured SHS exposure. The table also shows the inter-relationship between the SHS measures (legend).

In cross-sectional analysis, self-reported lower level SHS exposure (1–3 hours) was associated with poorer disease-specific QOL (mean score increment 3.6 points; 95% CI 0.4 to 6.7 points) and dyspnea (mean score increment 1.1 points; 95% CI 0.3 to 1.8) after controlling for covariates (Table 3). There was a suggestion that SHS exposure was related to COPD severity and physical health status, although the confidence intervals did not exclude the no effect level (Table 3).

The prospective analysis revealed that lower level SHS exposure (1–3 hours) was associated with poorer disease-specific QOL (mean score increase 3.4 points; 95% CI 0.3 to 6.5 points) and MRC dyspnea score after controlling for covariates (mean score increase 1.2 points; 95% CI 0.5 to 1.8 points) (Table 3). There was a suggestion that the self-reported SHS exposure was related to greater COPD severity and poorer physical health status, but the estimates were imprecisely estimated (Table 3).

### Directly measured SHS exposure and COPD-related outcomes

The highest urine cotinine tertile was associated, in cross-sectional multivariate analysis, with poorer COPD severity scores (mean score increment 4.7 points; 95% CI 0.6 to 8.9 points) and MRC dyspnea scores (score increment 1.0; 95% CI 0.4 to 1.7 points) (Table 4). There was a suggestion that the highest cotinine levels were related to greater impairment of physical health status scores (-5.6 points; 95% CI -12 to 1.0 points).

In longitudinal analysis, the highest urine cotinine tertile was associated with greater dyspnea (mean score increment 0.9 points; 95% CI 0.2 to 1.6). The highest cotinine level also appeared to be associated with worse COPD severity (mean score increment 4.7 points; 95% CI -0.1 to 9.4 points; p = 0.054), physical health status (-5.7 points; 95% CI -14 to 2.0 points), and disease-specific QOL (mean score increment 2.9 points (-0.16 to 5.9 points). The confidence intervals, however, did not exclude not effect. When compared to the standard deviation of each score, these changes achieved the minimal important difference criterion of 0.5 standard deviations (sd) or greater (i.e., COPD severity 0.7 sd, physical health status 0.5 sd, disease-specific QOL 0.6 sd, and dyspnea score 0.8 sd). Addition of *trans*-3'-hydroxycotinine/cotinine ratio to the multivariate model had essentially no impact on the estimates of SHS exposure effect (data not shown). The ratio itself was not associated with any outcome variable (p > 0.70 in all cases).

In contrast, there was no statistical relation between nicotine badge levels and any study outcome in either cross-sectional or prospective analysis (Tables 4 and 5).

## Discussion

We report the first study to assess the impact of objectively measured SHS exposure on a substantial cohort of adults with COPD. Despite the fact that they have chronic respiratory disease, SHS exposure was common among non-smoking adults with COPD. Although the results varied by measurement technique and type of analysis, SHS exposure was generally associated with greater COPD severity and poorer health status. The effect was observed in both cross-sectional and prospective analysis and appeared to be clinically meaningful, as judged against the criterion of the minimally important difference in health status indicators. Taking inter-individual differences in nicotine metabolism into account did not influence the estimates of SHS effect. These data suggest that passive smoking, in addition to direct personal cigarette smoking, may exert an important influence on outcomes in COPD.

Although the literature is small, previous studies suggest that SHS exposure may be a cause of new-onset COPD or impaired pulmonary function.[2,39-41] The effect of SHS exposure on persons with established COPD, however, has received very little study.[5] In a cohort of adults hospitalized for COPD, self-reported SHS exposure was a risk factor for re-hospitalization.[6] In another study based on the U.S. National Health Interview Survey, self-reported ETS exposure was related to a greater risk of "chronic respiratory disease exacerbation," defined as activity limitation or a physician visit due to asthma, chronic bronchitis, emphysema, or chronic sinusitis. The present findings add substantive additional evidence that SHS exposure is deleterious for patients with COPD.

The results differed somewhat depending on the method used to measure SHS exposure. For self-reported exposure, the overall pattern of results suggested a deleterious effect of SHS exposure on COPD-related health status, but the estimates were imprecise in many cases. This is probably attributable to the lower accuracy of self-reported SHS exposure. When urine cotinine was examined, which is an objective and specific measure of SHS exposure, the association between higher SHS exposure and poorer COPD-related health status was most clearly demonstrated. Personal badge nicotine levels, in contrast, were not associated with any health status variable. The different results for urine cotinine and personal badge nicotine levels may indicate that peak SHS exposure, rather than average SHS exposure, is more relevant to disease severity and health status in COPD. Personal badge data represent an integrated average exposure to nicotine during the time period that the badge is worn, whereas urinary cotinine data are more reflective of recent peak exposures. Alternatively, the personal nicotine badge measurements may have been subject to greater exposure misclassification than urine cotinine, because correct measurement depended on subjects reliably wearing the badge during all their daily activities (whereas urine cotinine was not). These methodologic differences likely also account for the low correlation among measures.

We used the standard epidemiologic definition of COPD, based on a self-reported physician diagnosis of chronic bronchitis, emphysema, or COPD [12-14]. This survey-based approach, supplemented by a mail-based sample acquisition strategy, allowed us to study a population-based sample of adults who resided throughout the United States, enhancing the generalizability of our findings. On logistical grounds, conducting spirometry among subjects who reside thousands of miles apart would be highly difficult, if not impossible. The use of self-reported physician-diagnosis, however, may have resulted in some misclassification of disease status. Previous work indicated that a similar survey-based definition of COPD had a high positive predictive value (78%) when validated using a blinded medical record review that included spirometry and radiographic studies. [42] Other work confirmed that a self-reported history of COPD is a strong predictor of airflow obstruction [43]. Our previous validation study indicated that nearly 9 out of 10 participants who had available spirometry data had objective evidence of airflow obstruction.[2] In sum, misclassification of COPD is not likely to bias our results; if present, such bias would likely be non-differential with respect to SHS exposure and reduce effect estimates towards the null value.

Although direct SHS exposure measurement is a more accurate method than self-report, it is more labor intensive and required a greater degree of commitment from study subjects. Reflecting these facts, not all eligible subjects participated in direct monitoring, which could have introduced selection bias. However, the similarity of participants and non-participants reduced the likelihood of this potential bias. In addition, incorporating sample weights that account for non-response into the analysis had a negligible impact on study results (data not shown). The other consequence of lower study participation is diminished statistical power, which resulted in decreased precision of effect estimates. In some cases, there appeared to be a negative effect of SHS exposure, but the 95% confidence intervals were wide and included no association. A larger sample size might have resulted in clearer evidence of SHS effects in these cases and would be required to detect smaller effects

Other limitations include the potential confounding effects of direct cigarette smoking. We attempted to reduce this confounding effect by limiting the analysis to persons who indicated no current smoking and had urine cotinine levels below a cut-point usually associated with active smoking. We also statistically controlled for a past history of cigarette smoking in multivariate analysis. Nonetheless, we cannot fully exclude confounding by active smoking. And finally, although direct SHS exposure measurement eliminated the bias inherent in self-reported exposure, a bias termed the "healthy passive smoker effect" can still occur, meaning that more severely affected COPD patients may selectively avoid SHS exposure, attenuating the observed association between exposure and asthma health outcomes.

## Conclusion

There is no question that cigarette smoking is the dominant risk factor for COPD and is the most important factor driving the progression of airflow obstruction. Our results implicate SHS exposure as another important factor influencing disease severity and health status in this health condition. Because SHS is a modifiable risk factor, clinicians should assess SHS exposure in their patients and counsel its avoidance. In public health terms, the effects of SHS exposure on this vulnerable subpopulation provide a further rationale for laws prohibiting public smoking.

## Competing interests

The author(s) declare that they have no competing interests.

## Authors' contributions

MDE designed and carried out the direct SHS monitoring study, performed the analysis, and wrote the paper; JB participated in study design and writing of the manuscript; EY participated in study design and manuscript writing; PK contributed to study design and manuscript writing; NB performed the urine cotinine and hydroxycotinine analyses and contributed to the manuscript writing; PB contributed major effort to the overall study design and contributed to the analysis and manuscript writing.

## Pre-publication history

The pre-publication history for this paper can be accessed here:



## Figures and Tables

**Table 1:** Characteristics of adult non-smoking participants with COPD

Characteristic	Overall non-smoking COPD cohort (n = 152)	Participants in direct SHS exposure assessment study	
		Completed initial assessment (n = 77)	Completed baseline and 1 year follow-up assessment (n = 68)
Age (yrs)	64.4 (6.0)	65.0 (6.4)	64.8 (6.2)
Male gender (%)	55 (36%)	30 (39%)	27 (40%)
Race-ethnicity (white, non-hispanic)	138 (91%)	72 (94%)	65 (96%)
Educational attainment			
High school or less	75 (49%)	33 (43%)	31 (46%)
Some college	44 (29%)	26 (34%)	20 (29%)
College graduate or graduate degree	33 (22%)	18 (23%)	17 (25%)
Past smoking history (%)	109 (72%)	51 (66%)	45 (66%)
COPD severity score	8.2 (6.3)	8.3 (6.6)	8.2 (6.7)
SF-12 physical component summary score	35.0 (11.8)	34.8 (11.7)	35.4 (11.6)
Airways Questionnaire 20 (AQ-20)	8.6 (5.0)	8.5 (4.7)	8.5 (4.6)
Modified MRC dyspnea score	2.5 (1.2)	2.5 (1.2)	2.5 (1.2)

Higher scores on COPD severity score, Airways Questionnaire 20, and modified MRC dyspnea score = poorer health status; higher score on SF-12 Physical Component Summary Score = better health status. There were no statistical differences between non-smoking COPD participants who did and did not complete initial direct SHS assessment (p > 0.15, all cases) (data for those who did not complete initial direct SHS assessment are not shown separately); also no differences between those who completed initial direct SHS assessment who did and did not complete 1 yr follow-up (p > 0.05, all cases).

**Table 2:** Distribution of SHS exposure among non-smokers with COPD by self-report and direct exposure monitoring

Measure	N	Proportion with any self-reported exposure or detectable urine/badge level	SHS exposure among those with any exposure		
			
			25^th ^%tile	Median	75^th ^%tile
Self-report, past 7 days (hrs)	77	20 (26%)	1	2	7
Urine cotinine (ng/ml)	77	69 (90%)	0.092	0.20	0.81
First tertile*	26	14 (54%)	0	0.054	0.069
Second tertile	26	26 (100%)	0.12	0.16	0.22
Third tertile	25	25 (100%)	0.75	1.54	3.86
Personal nicotine badge (μg/m^3^)	77	64 (83%)	0.096	0.22	0.63
First tertile*	27	14 (52%)	0	0.023	0.061
Second tertile	24	24 (100%)	0.12	0.14	0.22
Third tertile	26	26 (100%)	0.45	0.86	1.53

*Based on the distribution, urine cotinine and personal badge nicotine levels were divided into tertiles for statistical analysis. Although lowest tertile contained some subjects with detectable values, these were very low values quantitatively.

Among 77 subjects with all three measures available, the Spearman rank correlation between self-reported SHS exposure and urine cotinine was 0.28 (p = 0.013); between self-reported exposure and badge nicotine level was 0.06 (p = 0.57); between urine cotinine and badge nicotine was 0.41 (p = 0.0002). When tertile of urine cotinine and nicotine badge were examined as categories, the Kappa was 0.33 (95% CI 0.16 to 0.50).

**Table 3:** Impact of self-reported SHS exposure (past 7 days) on COPD-related health status among 77 adults with COPD: cross-sectional and prospective analysis

SHS exposure, past 7d	COPD severity		Physical health status (SF-12)		Disease-specific QOL		Dyspnea	
	Bivariate	Multivariate	Bivariate	Multivariate	Bivariate	Multivariate	Bivariate	Multivariate
**Cross-sectional analysis (n = 77)**								
None	Referent	Referent	Referent	Referent	Referent	Referent	Referent	Referent
1–3 hrs	1.0 (-3.2 to 5.2)	1.7 (-2.7 to 6.2)	-0.6 (-8.1 to 6.9)	-5.9 (-12.8 to 1.0)^‡‡^	2.4 (-0.7 to 5.5)	**3.6 (0.4 to 6.7)**	0.5 (-0.3 to 1.2)	**1.1 (0.3 to 1.8)**
≥4 hrs	4.1 (-0.91 to 9.0)^†^	3.8 (-1.2 to 8.8)	-1.5 (-10.4 to 7.4)	-1.3 (-9.1 to 6.6)	1.0 (-2.8 to 4.8)	1.2 (-2.4 to 4.7)	0.2 (-0.7 to 1.1)	0.2 (-0.6 to 1.0)
**Impact of baseline SHS exposure on prospective outcomes at 1 year (n = 68)**								
None	Referent	Referent	Referent	Referent	Referent	Referent	Referent	Referent
1–3 hrs	3.2 (-1.2 to 7.7)	3.6 (-1.1 to 8.2)	-5.7 (-13.2 to 1.8)	-7.2 (-14.9 to 0.5) ^‡^	2.5 (-0.9 to 5.8)	**3.4 (0.3 to 6.5)**	**0.9 (0.2 to 1.7)**	**1.2 (0.5 to 1.8)**
≥4 hrs	4.9 (-0.2 to 9.9)*	4.1 (-1.1 to 9.2)^††^	-6.5 (-15.0 to 2.1)	-4.9 (-13.5 to 3.7)	2.6 (-1.2 to 6.4)	1.6 (-1.8 to 5.1)	0.4 (-0.4 to 1.3)	0.2 (-0.5 to 1.0)

Values are mean score change compared to lowest exposure tertile (referent group) and 95% confidence interval. The lower and higher SHS exposure groups were defined based on the median among those with any exposure. Multivariate analysis controls for age, sex, race, educational attainment, and past smoking history. Physical health status was measured by the SF-12 physical component summary score; Disease-specific QOL was measured by the Airways Questionnaire 20 (AQ-20); Dyspnea was measured by the modified MRC Dyspnea Scale. Higher scores = worse status, except for SF-12 physical score. There were 57 subjects, 12 subjects, and 8 subjects in the none, 1–3 hrs, and ≥4 hrs/week exposure groups, respectively.

Boldface = p < 0.05.

*p = 0.06 ^‡^P = 0.07 ^‡‡^p = 0.09 ^†^p = 0.11 ^††^p = 0.12

**Table 4:** Cross-sectional association between directly measured SHS exposure and COPD-related health status (n = 77).

SHS exposure	COPD severity		Physical health status (SF-12)		Disease-specific QOL		Dyspnea	
	Bivariate	Multivariate	Bivariate	Multivariate	Bivariate	Multivariate	Bivariate	Multivariate
**Urine cotinine**								
1^st ^tertile	Referent	Referent	Referent	Referent	Referent	Referent	Referent	Referent
2^nd ^tertile	1.2 (-2.5 to 4.8)	0.2 (-3.6 to 3.9)	-2.3 (-8.9 to 4.2)	0.4 (-5.6 to 6.5)	-0.4 (-3.2 to 2.4)	-0.7 (-3.5 to 2.0)	-0.1 (-0.7 to 0.6)	-0.3 (-0.9 to 0.3)
3^rd ^tertile	**5.4 (1.4 to 9.3)**	**4.7 (0.6 to 8.9)**	**-7.8 (-14.9 to -0.7)**	-5.6 (-12 to 1.0)	2.9 (-0.16 to 6.0)	2.3 (-0.7 to 5.4)	**1.1 (0.5 to 1.8)**	**1.0 (0.4 to 1.7**)
**Nicotine badge**								
1^st ^tertile	Referent	Referent	Referent	Referent	Referent	Referent	Referent	Referent
2^nd ^tertile	0.2 (-3.6 to 3.9)	0.6 (-3.2 to 4.4)	3.0 (-3.7 to 9.8)	2.7 (-3.4 to 8.8)	-1.6 (-4.5 to 1.4)	-1.7 (-4.6 to 1.2)	-0.2 (-0.8 to 0.5)	-0.2 (-0.8 to 0.4)
3^rd ^tertile	-1.4 (-5.3 to 2.5)	-1.1 (-5.0 to 2.8)	3.2 (-3.8 to 10.1)	1.9 (-4.3 to 8.2)	-2.1 (-5.1 to 0.9)	-1.6 (-4.5 to 1.3)	-0.2 (-0.9 to 0.5)	-0.1 (-0.7 to 0.5)

All values are mean score change compared to lowest exposure tertile (referent group) and 95% confidence interval. Multivariate analysis controls for age, sex, race, educational attainment, and past smoking history. Physical health status was measured by the SF-12 physical component summary score; Disease-specific QOL was measured by the Airways Questionnaire 20 (AQ-20); Dyspnea was measured by the modified MRC Dyspnea Scale. Higher scores = worse status, except for SF-12 physical score. Boldface = p < 0.05.

**Table 5:** Prospective impact of directly measured SHS exposure on COPD-related health status at 1 year follow-up (n = 68)

SHS exposure	COPD severity		Physical health status (SF-12)		Disease-specific QOL		Dyspnea	
	Bivariate	Multivariate	Bivariate	Multivariate	Bivariate	Multivariate	Bivariate	Multivariate
**Urine cotinine**								
1^st ^tertile	Referent	Referent	Referent	Referent	Referent	Referent	Referent	Referent
2^nd ^tertile	2.8 (-1.2 to 6.7)	1.9 (-2.3 to 6.0)	0.4 (-6.3 to 7.0)	2.6 (-4.2 to 9.4)	-0.3 (-3.1 to 2.5)	-1.4 (-4.1 to 1.3)	0.1 (-0.8 to 0.6)	-0.2 (-0.8 to 0.4)
3^rd ^tertile	**5.8 (1.5 to 10.2)**	4.7 (-0.1 to 9.4)*	**-7.8 (-15 to -0.6)**	-5.7 (-14 to 2.0)	**4.5 (1.3 to 7.6)**	2.9 (-0.16 to 5.9)^†^	**1.2 (0.5 to 1.9)**	**0.9 (0.2 to 1.6)**
**Nicotine badge**								
1^st ^tertile	Referent	Referent	Referent	Referent	Referent	Referent	Referent	Referent
2^nd ^tertile	0.5 (-3.6 to 4.6)	1.0 (-3.3 to 5.3)	0.1 (-6.8 to 6.9)	-0.2 (-7.2 to 6.9)	-0.2 (-3.1 to 2.8)	0.3 (-2.5 to 3.0)	-0.1 (-0.8 to 0.6)	0 (-0.7 to 0.6)
3^rd ^tertile	-0.7 (-4.8 to 3.5)	-0.3 (-4.6 to 4.0)	-1.1 (-8.1 to 5.9)	-2.1 (-9.2 to 5.0)	-0.1 9–3.1 to 2.9)	0.9 (-1.9 to 3.7)	0 (-0.6 to 0.7)	0.2 (-0.5 to 0.8)

All values are mean score change compared to lowest exposure tertile (referent group) and 95% confidence interval. Multivariate analysis controls for age, sex, race, educational attainment, and past smoking history. Physical health status was measured by the SF-12 physical component summary score; Disease-specific QOL was measured by the Airways Questionnaire 20 (AQ-20); Dyspnea was measured by the modified MRC Dyspnea Scale. Higher scores = worse status, except for SF-12 physical score.

Boldface = p < 0.05.

*p = 0.054

^†^p = 0.063
